# Detection system− and strain−dependent diversity of de novo [PSI+] prion generation and phenotypes in *Saccharomyces cerevisiae*

**DOI:** 10.71150/jm.2506009

**Published:** 2025-09-18

**Authors:** Moonil Son

**Affiliations:** 1 Department of Microbiology, Pusan National University, Busan 46241, Republic of Korea; 2 Microbiological Resource Research Institute, Pusan National University, Busan 46241, Republic of Korea

**Keywords:** yeast prion [PSI+], nonsense-suppression

## Abstract

Yeast prion [PSI+], an amyloid form of the translation termination factor Sup35p/eRF3, causes translational stop codon readthrough by sequestering functional Sup35p. This unique phenotype may be analyzed via [PSI+]−suppressible nonsense alleles, and has greatly contributed to the advancement in yeast prion research. For comparing canonical reporters, like chromosomal *ade1−14* or *ade2−1*, and plasmid-borne *ura3−14*, the de novo generation and characteristics of [PSI+] was investigated across common yeast laboratory strains (BY4741, 74D−694, and 779−6A). The results showed significant variability in [PSI+] induction frequency among strains. [PSI+] was successfully induced in BY4741 and frequently in 74D−694 (via Ade^+^ selection), but not in 779−6A. Notably, [PSI+] clones, even from identical genetic backgrounds, displayed vastly different nonsense suppression phenotypes depending on the reporter allele used; resulting in diverse growth patterns and suppression levels. Quantitative analyses revealed that prion seed counts fluctuated significantly based on the detection allele and observed phenotype. Furthermore, Sup35p aggregate visualization revealed distinct structural patterns between BY4741 and 74D−694, indicating strain-specific differences. Transferring [PIN+] prion variants from different strains into a common [psi−][pin−] background yielded similar [PSI+] inducibility and seed numbers, suggesting that the observed phenotypic and quantitative diversities of [PSI+] prions stem primarily from the interplay between the specific reporter detection system and the host strain's genetic background rather than solely from inherent differences in the initial [*PIN*+] prion or fundamental changes in the [PSI+] protein itself. This study underscores the crucial need to consider both the detection methodology and host genetic context for accurate prion variant characterization.

## Introduction

Prions are proteinaceous infection particles that were considered mammalian and human pathogens for a long time after the first experimental evidence of their existence (Prusiner, 1982). However, since the discovery of the prions [PSI+] and [URE3] in the model microorganism *Saccharomyces cerevisiae*, several more prions were reported in budding yeast, and prions were searched in other microbes, such as the fission yeast *Schizosaccharomyces pombe*, the filamentous fungus *Podospora anserina*, and even bacteria (Alberti et al., 2009; Coustou et al., 1997; Derkatch et al., 2001; Fleming et al., 2019; Sharma et al., 2024; Son et al., 2023; Sondheimer and Lindquist, 2000; Wickner, 1994; Yuan and Hochschild, 2017). Along with these discoveries, the ease of manipulation of yeast and fungal genetics has led to pioneering studies on prions and host physiology at the cellular level (Son, 2024, 2025).

Yeast [PSI+] is the prion form of the translation release factor Sup35p/eRF3. This factor interacts with Sup45p/eRF1, which is required for stop codon recognition, and. together, they play a role in translation termination (Stansfield et al., 1995). The structural conversion of Sup35p into the [PSI+] prion form results in the inactivation of its own function; thus, translation termination does not occur, neither by readthrough of the termination codon or premature stop codon (nonsense) suppression. Based on the [PSI+] prion phenotype, nonsense-containing alleles have been introduced into yeast strains to detect or score [PSI+] prions. Combinations of *SUQ5* (a weak tRNA suppressor) with alleles *ade2−1* or *ade1−14* (with UAA and UGA stop codons inserted in their ORFs, respectively) have been used as [PSI+] prion-reporter systems (Chernoff et al., 1995; Cox, 1965; Inge-Vechtomov et al., 1988; Liebman et al., 1975). For example, in these *ade* nonsense allele systems, cells with [PSI+] show a typical white color on rich media (YPD) or in media with limited adenine; they are even able to grow in media without supplementary adenine. In contrast, cells without the prion, i.e., [psi−], are red colored on rich media and not able to grow on media lacking adenine. In addition to chromosomally integrated nonsense alleles, the [PSI+]−suppressible nonsense *URA3* gene, inserted into a plasmid, has been used to screen strains with an *ura3* mutation and without *ade2−1* or *ade1−14* mutations, such as S288C and its derivatives (Manogaran et al., 2006). These [PSI+]−suppressible nonsense alleles not only facilitate in−depth studies about the [PSI+] prion by allowing the selection of [PSI+] or [psi−] cells but also provide a plasmid-based [PSI+] scoring system that may be used in a wide range of yeast strains (Son, 2025).

A unique biological property among human, mammalian, and yeast prions is that a single prion protein sequence can give rise to multiple distinct “prion variants” or “strains” (Bradley et al., 2002; Derkatch et al., 1996; King, 2001; Schlumpberger et al., 2001; Tanaka et al., 2004). These prion variants are usually stably propagated, with amyloid filaments templating new monomers to adopt the same conformation as that of the existing amyloid molecules when they join both ends of the filaments (Wickner et al., 2015). In yeast, prion variants were first classified in accordance with the strength of cell phenotypes as “strong” and “weak,” referring to nonsense suppression efficiency of individual [PSI+] isolates or as “lethal” and “mild,” when referring to the degree to which these prions inhibit cell growth or affect cell viability, respectively (Derkatch et al., 1996; Nakayashiki et al., 2005). Later, numerous prion variants were reported to differ in their propagation stability: the degree of transmission from mother to daughter cells during mitosis; chaperone dependence: the ability to propagate in the presence (usually overproduction) or absence of specific chaperones, such as Hsp104; and transmission barrier crossing ability: the capacity to transmit across inter- or intra-species barriers, that is, between different species or within the same species but with different genetic backgrounds, respectively (Wickner et al., 2019).

A cumulus of observations and experimental evidence about prion variants originated the “prion cloud” model, which proposes that a “cloud” of prion variants segregate and mutate at a certain frequency as cells grow. The model signifies the dynamic and heterogeneous nature of prion populations within a cell, suggesting that prions are not static entities, but rather evolving populations, emphasizing the need for prion-handling systems (described below) to be sufficiently robust to handle this heterogeneity (Bateman and Wickner, 2012, 2013). This genetics-based prion variant concept is supported by extensive solid-state NMR studies. These structural approaches have confirmed that yeast prion amyloids (Sup35p, Ure2p, and Rnq1p) have a folded, parallel, in−register β-sheet architecture (Baxa et al., 2007; Shewmaker et al., 2006; Wickner et al., 2008). These resolved structures provide not only an explanation on how these proteins can template their own structure/conformation, as identical residues can favorably interact in this arrangement, ensuring faithful propagation of conformational information, but also an explanation on how a single protein can lead to multiple distinct “prion variants” with stable propagation ability (Tycko, 2014; Wickner et al., 2015).

Most prion variants are pathogenic and severely hinder growth or even kill host cells (Nakayashiki et al., 2005). These pathogenic variants are rarely found in wild strains, suggesting that prions have detrimental effects on cells, and that cells have evolved robust anti-prion systems to counteract them. A series of studies has revealed that yeast has multi-layered anti-prion systems that inhibit the emergence of prion variants, cure those that have already been developed, and mitigate their pathogenicity (Son et al., 2023; Son and Wickner, 2022). For instance, Hsp104 is essential for most yeast prion propagation, but also functions as an anti-prion system by curing many [PSI+] variants arising in its absence (Gorkovskiy et al., 2017). Btn2p and Cur1p are also known to cure most newly formed [URE3] prion variants in *btn2*Δ*cur1*Δ strains by restoring these proteins (Kryndushkin et al., 2008; Wickner et al., 2014). Nonsense-mediated mRNA decay (NMD) factors (Upf1, 2, and 3p), required for the degradation of aberrant mRNA, can cure newly formed prion variants, possibly by directly binding to prion-forming Sup35p monomers or interfering with fibril elongation (Son and Wickner, 2018). Additionally, Siw14p, which is involved in inositol pyrophosphate metabolism, can cure [PSI+] prion variants (e.g., [PSI+]) by modulating the levels of specific inositol polyphosphate signaling molecules that influence prion dynamics (Wickner et al., 2017). These NMD factors and Siw14p were found in a yeast genetics-based screening, and the combined results suggested the dual functions of each anti-prion component: mRNA quality control of NMD factors or metabolic regulation of Siw14p, respectively, while they both have the ability to counteract [PSI+] prions derived from their own functions.

The Ribosome−associated complex (RAC), consisting of Ssb1/2p (Hsp70)-Zuo1p (Hsp40)-Ssz1p (Hsp70), was previously known for preventing misfolding of newly synthesized polypeptides; the lack of any of them leads to increased [PSI+] prion frequencies (Amor et al., 2015; Chernoff et al., 1999; Gautschi et al., 2001, 2002; Kiktev et al., 2015; Pfund et al., 1998; Rakwalska and Rospert, 2004; Yan et al., 1998). Moreover, most [PSI+] prion isolates formed in RAC component-deletion mutants were cured after the restoration of each of the components, even when expressed at normal levels (Son and Wickner, 2020). Although the previously mentioned anti-prion systems are in charge of prion variants arising in the absence of each of such systems, most [PSI+] prion variants arising in anti-prion system multiple deletion mutants (i.e., *ssz1*Δ*upf1*Δ*hsp104^T160M^* strain, *suh*) were cured through the cooperation of the systems (Son and Wickner, 2022). Taken together, prion variants can be categorized based on their sensitivity to anti-prion systems, with most prion isolates being sensitive to multiple systems.

In addition, a series of studies on [PSI+] prion variants categorized by their propagation diversity have been conducted using well-established prion variant typing (Huang and King, 2020; Huang et al., 2021; King, 2025; Yu and King, 2019). Twenty-three [PSI+] prion variants of WT Sup35p with different propagation abilities (19 novel variants and 4 previously documented variants: VH, VK, VL, and W8) were obtained and confirmed to be unique and not a composite of other variants (Huang and King, 2020). Although a recent study on the [PSI+] prion variants arising in a *suh* triple mutant of the 74D−694 yeast strain showed contrary results to those of previous reports, it facilitated an in-depth alternative analysis on how *ssz1*Δ and *upf1*Δ mutations can independently contribute to or facilitate prion propagation. This study also showed that [PSI+] prion variants are stringently dependent on the appropriate activities of Hsp104 disaggregase, and that these variants can be categorized based on their sensitivity to Hsp104 activity levels (King, 2025). Understanding these yeast prion variants has significant implications for research on human amyloidoses, such as Alzheimer's and Parkinson's diseases. However, yeast prion variants are generally isolated using specific detection systems, such as the nonsense allele for [PSI+], and the yeast strains preferred by each research group. Therefore, a comparative analysis of yeast prion variants isolated through different detection systems and from different strain backgrounds is needed to connect all those previously conducted studies. In this study, we used three representative conventional laboratory yeast strains (BY4741, 74D−694, and 779−6A), widely used for [PSI+] prion studies, and isolated various [PSI+] prion variants with different [PSI+]-suppressible alleles (*ade1−14* or *ade2−1* and *ura3−14*). Furthermore, we showed that almost none of the [PSI+] variants isolated using one detection system overlap with those obtained using other systems in their nonsense suppression phenotypes.

## Materials and Methods

### Nomenclature

Yeast prions are shown in brackets to indicate they are non-chromosomal genes, e.g., [PSI+] and [PIN+]. Phenotypes of yeast strains were marked with superscripted +/−. Dropout (DO) media with corresponding amino acid(s) was designated with − and three letter codes.

### Strains, plasmid, and media

This study utilized yeast strains and plasmids detailed in Table 1. All media preparations followed previously established protocols (Sherman, 1991). To induce the expression of Sup35p NM under the control of the *GAL1* promoter, cells were grown in media containing 2% galactose and 2% raffinose, a method consistent with prior research (Son and Wickner, 2018). The parental strains, including those with a BY4741 background that carry the *ade1−14* allele, were derivatives from earlier studies (Son and Wickner, 2018). For scoring [PSI+] using the suppressible *ura3−14* mutation and for inducing prion formation, the pM6/p1520 plasmid was routinely employed. This plasmid is a centromeric vector containing the *LEU2* marker, the *ura3−14* allele, and the *GAL1* promoter driving *SUP35 NM* expression. To ensure the consistent presence of the pM6 plasmid throughout all [PSI+] scoring experiments, strains and colonies were maintained on media lacking leucine.

### [PSI+] seed measurement

**Fig. 1.**
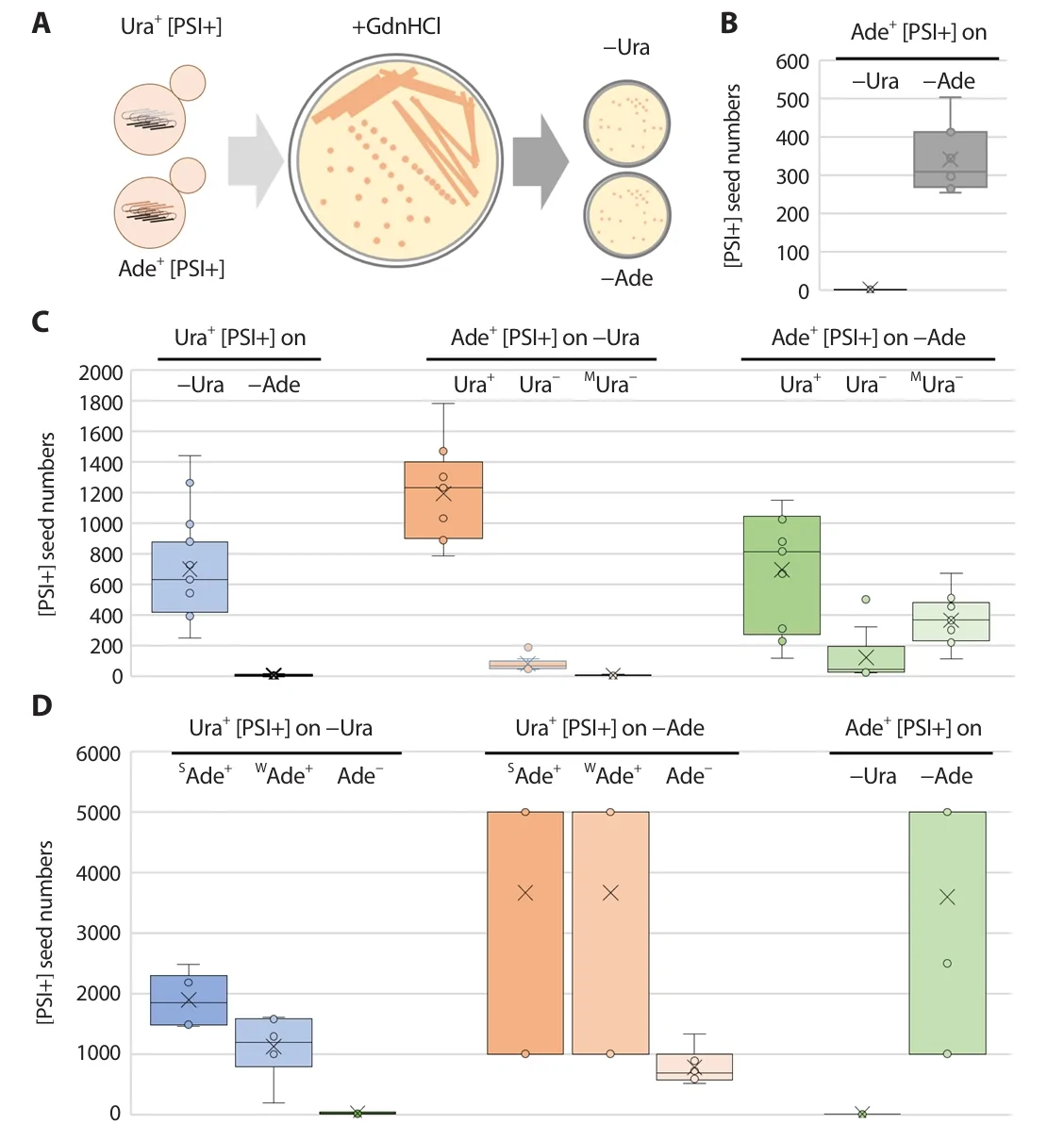
The seed number measurement of [PSI+] isolates. (A) Experimental procedure of the measurement of [PSI+] prion seed number. Ura^+^ or Ade^+^ [PSI+] isolates in each strain were streaked on 5 mM GdnHCl containing media. Separated single colonies were resuspend and further spread on the media lacking uracil (−Ura) or adenine (−Ade). The number of arising Ura^+^ or Ade^+^ single colonies were regarded as pre−existing [PSI+] prion seeds in each strain. (B) The seed number of Ade^+^ [PSI+] isolates in 779−6A were determined (n = 12). (C) The seed numbers of [PSI+]s in BY4741 were counted (n = 12). Ura^+^ [PSI+] isolates were determined on different media (light blue, left panel). Ade^+^ [PSI+] isolates with three different uracil auxotrophic phenotypes (Ura^+^, Ura^−^, and ^M^Ura^−^) seed numbers were confirmed on the media lacking uracil (−Ura, light orange, middle panel) or adenine (−Ade, light green, right panel). (D) The seed numbers of [PSI+]s in 74D−694 were counted (n = 12). Ura^+^ [PSI+] isolates with three different adenine auxotrophic phenotypes (^S^Ade^+^, ^W^Ade^+^, and Ade^−^) seed numbers were confirmed on the media lacking uracil (−Ura, light blue, left panel) or adenine (−Ade, light orange, middle panel). Ade^+^ [PSI+] isolates were determined on different media (light green, right panel). Arising Ura^+^ or Ade^+^ ([PSI+]) clones were tested for GdnHCl curability using transient growth on plates containing 5 mM guanidine.

Following the method described by Cox et al. (2003), freshly grown [PSI+] cells were streaked for single colonies on YPAD medium supplemented with 5 mM Guanidine Hydrochloride (GdnHCl). Individual colonies, along with the underlying agar block, were then suspended in distilled water and plated onto media lacking either uracil or adenine. Colonies that exhibited the Ura^+^ or Ade^+^ phenotype were counted, with each such colony presumed to represent a [PSI+] prion "seed" present in the cell that initiated that colony (Fig. 1A). For each yeast strain tested, at least ten individual colonies underwent this analysis. A subset of the Ura^+^ or Ade^+^ colonies obtained were subsequently checked for their guanidine curability to confirm that the observed phenotype was indeed due to the [PSI+] prion.

### Cytoduction

The *kar1* mutation results in a failure of nuclear fusion following cytoplasmic fusion during the yeast mating process. Consequently, the cytoplasms of the resulting "daughter" cells are a mixture of those from the two parental strains. This phenomenon is leveraged in cytoduction, a widely used technique for transferring cytoplasmic genes from a donor strain to a recipient strain.

In this process, the transfer of mitochondrial DNA (ρ), indicated by the ability to grow on glycerol (ρ^+^), is typically used as a marker for successful cytoplasmic transfer. The donor strain is ρ^+^ (glycerol−utilizing), while the recipient strain is rendered ρ^o^ (lacking mitochondrial DNA) by growth in ethidium bromide-containing media. Both donor (carrying its own [PIN+] prion) and recipient ([pin−], previously cured of prions by GdnHCl treatment) strains carried the pM6 plasmid. For cytoduction, donor (ρ^+^) and recipient (ρ^o^) cells were mixed in distilled water, with approximately a three-fold excess of donor cells, and spotted onto a YPAD plate to facilitate mating. Following incubation at 30°C, the cell mixture was streaked for single colonies on media designed to select against the donor. After a further three days of incubation at 30°C, individual colonies were replica-plated onto YPG (glycerol−containing media, selective for ρ^+^ cells), media selective for diploids, and media lacking uracil. Clones that grew on YPG but failed to grow on diploid selection media were identified as cytoductants (i.e., cells that received cytoplasm but not a fused nucleus from the donor). The successful transfer of [PIN+] prions from the donor was additionally verified through microscopic observation and by testing for [PSI+] prion inducibility in the cytoductants.

## Results

### De novo [PSI+] generation differs among laboratory yeast strains

Sup35p overproduction (either the Sup35p N or NM domains) has been reported to induce [PSI+] prion generation (Derkatch et al., 1996; Wickner, 1994). Therefore, [PSI+] was induced in three representative yeast strains that are widely used for [PSI+] prion studies for further analysis. To compare [PSI+] detectability among [PSI+]−suppressible nonsense alleles, such as *ade1−14* (on chromosome of BY4741 derivatives and 74D−694) or *ade2−1* (on chromosome of 779−6A), strains were transformed with a centromeric plasmid pM6/p1520 (Table 2; Son and Wickner, 2018, 2020, 2022; Wickner et al., 2017), harboring the nonsense allele *ura3−14* (Manogaran et al., 2006), and the [PSI+] prion forming domain (NM) of Sup35p under the control of the galactose-inducible *GAL1* promoter. Strains were grown in galactose and raffinose for 2 days, cells were serially diluted and plated on media lacking uracil (−Ura) and adenine (−Ade) to select Ura^+^ and Ade^+^ [PSI+] clones. For further analysis, the colonies of each strain were tested for prion curability by transient growth on media containing 5 mM guanidine hydrochloride (GdnHCl) (Jung et al., 2002; Jung and Masison, 2001; Tuite et al., 1981).

The frequency of Ura^+^ [PSI+] clones in 74D−694 was slightly lower than that in BY4741, whereas Ura^+^ colonies in 779−6A were not curable by GdnHCl, indicating that the arising colonies were not [PSI+] (Table 3). Besides, Ade^+^ [PSI+] clones appeared most frequently in 74D−694 strain cells on −Ade plates (Table 3). Ade^+^ colonies were curable in all cases.

### Nonsense suppression phenotypes differ depending on the reporter alleles and strains used

Most [PSI+] clones isolated using a certain nonsense allele stably propagated in their own background. However, they sometimes showed dissimilar propagation phenotypes when a different nonsense allele is used, even in the same background (King, 2025; Son and Wickner, 2018; Yu and King, 2019). To test [PSI+]–suppressible phenotypes using two different nonsense allele-based detection systems, GdnHCl–curable Ura^+^ or Ade^+^ [PSI+] clones (Table 3) were streaked on non-selective media and replica plated on selective media (–Ura and –Ade). In the BY4741 background, none of the Ura^+^ [PSI+] clones grew on media lacking adenine (Ade^−^ phenotype), while Ade^+^ [PSI+] clones showed distinct groupable phenotypes on media lacking uracil (Ura^+^: 6/18, Ura^−^: 6/18, and mixed but mostly Ura^−^: 6/18; Table 4). In the 74–D694 background (*ade1−14*), in contrast, Ura^+^ [PSI+] clones showed groupable growth phenotypes on –Ade plates (Strong Ade^+^: 3/18, Weak Ade^+^: 6/13, and Ade^−^: 4/13). Though most Ade^+^ [PSI+] clones were Ura^−^, approximately 20% of the subclones of a fraction of these clones (12/18) showed an unstable Ura^+^ phenotype (Table 4). In the 779–6A strain background (ade2–1), isolatable Ade^+^ [PSI+] clones had a uniform phenotype (Table 4). We also tested strains L2892 (with a strong [PSI+] variant) and L2885 (with a weak [PSI+] variant), which were previously isolated using the *ade1−14* allele and have been conventionally used in many [PSI+] experiments (Mathur et al., 2009). L2892 showed even frequencies of Ura^+^ and Ura^−^ phenotypes, whereas L2885 showed a uniform Ura^−^ phenotype on –Ura media (Table 4).

### Seed numbers of [PSI+] variants isolated by different detection systems

The number of prion seeds (propagons) or infectious prion particles in each prion-carrying strain was calculated using GdnHCl, which is a specific inhibitor of the Hsp104 chaperone that is required for prion propagation via amyloid filamentation (Cox et al., 2003). Millimolar treatment with GdnHCl halts amyloid filamentation, and new filaments are no longer generated. Therefore, the number of colonies arising on prion-selectable media after the spreading of a single colony on a GdnHCl-containing medium reflects the number of preexisting seeds in a prion-carrying strain (Fig. 1A).

The number of seeds of Ade^+^ [PSI+] clones in the 779–6A background were approximately 350 on –Ade plates, but 0 on –Ura plates, indicating that the *ura3–14* allele could not detect the seeds of the Ade^+^ [PSI+] clones (Fig. 1B).

In the BY4741 background, Ura^+^ [PSI+] clones (with a uniform Ade^−^ phenotype) showed seed numbers that ranged between 400 and 900 when detected on –Ura plates, but that were undetectable by the *ade1–14* allele. Regarding Ade^+^ [PSI+] clone seed numbers calculated on –Ura media, only those with a Ura^+^ phenotype showed reliable seed numbers, which varied between 900 and 1,400 (Fig. 1B). The numbers of Ade^+^ [PSI+] clones on –Ade media correlated with their Ura^+^/Ura^−^ phenotypes: 300–1,000 seeds for Ura^+^ clones, less than 200 seeds for Ura^−^ clones, and 200–500 seeds in mixed but mostly Ura^−^ clones (Fig. 1C).

In the 74–D694 background, Ura^+^ [PSI+] clone seed numbers were calculated on –Ura media and correlated with their Ade^+^/Ade^−^ phenotypes, therefore, the seed number increased as the strength of Ade phenotype increased (Fig. 1D). When using the *ade1–14* allele (on –Ade media) for detecting Ura^+^ or Ade^+^ [PSI+] clones, seed numbers showed wide fluctuations (10^3^ or over 10^3^) and only the seed number of Ura^+^ [PSI+] clones with an Ade^−^ phenotype could be measured: 600–1,000 seeds. The number of seeds of Ade^+^ [PSI+] clones on –Ade media showed the same type of fluctuation, and almost no seeds were obtained on –Ura media (Fig. 1D).

### Aggregation of [PSI+] prion variants in different strain backgrounds

**Fig. 2.**
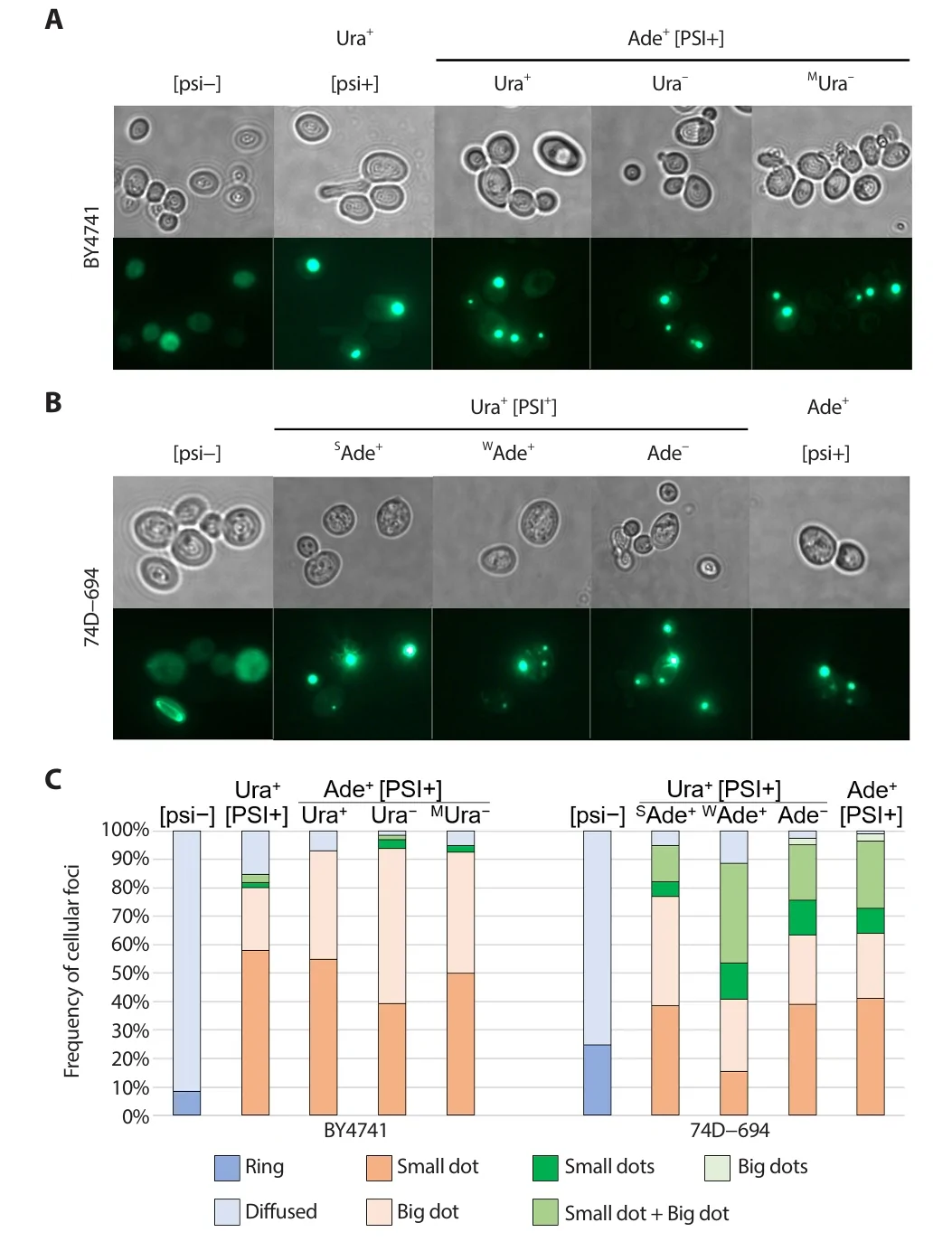
Sup35 NM−GFP aggregates in [PSI+] isolates. [psi−] strain and original Ura^+^ or Ade^+^ [PSI+] isolates with different phenotypes (left to right) were transformed with a *CEN* plasmid pM107 encoding Sup35 NM-GFP controlled by the *ADH1* promoter. The original isolates and their phenotypes examined is shown above the image. After Incubation for at least 16 h in 2% (wt/vol) glucose minimal medium, Sup35 NM-GFP aggregates or diffused GFP signal were observed using fluorescence microscopy (Imager M2, Zeiss). (A) Sup35 NM−GFP aggregates in BY4741 strain background. (B) Sup35 NM−GFP aggregates in 74D−694 strain background. (C) The frequencies of cells with different foci in BY4741 and 74D−694 strain were determined. The frequencies of cells with aggregates were scored in 100−300 cells. [psi−] strain and the original isolates and their phenotypes examined is shown above the bar.

The expression of GFP-tagged prion proteins is used to visualize preexisting [PSI+] prion aggregates in cells (Son and Wickner, 2018, 2020). To investigate whether the discrepancy in prion aggregate formation depended on strain background, isolation method, or phenotype, Sup35 NM-GFP was transiently overexpressed in each strain (Fig. 2). [psi–] strains of both backgrounds consistently showed a diffuse GFP signal (no prion aggregates) and rare formation of a ring structure (a sign of ongoing prion generation). In contrast, [PSI+]-carrying strains faithfully showed GFP foci (Fig. 2A and 2B; Mathur et al., 2010; Son and Wickner, 2018, 2020)

The formation of prion aggregate structures has been reported to be affected by prion variants with different characteristics, but without any chromosomal mutation (wild-type strain) (Huang et al., 2013; Zhou et al., 2001). To investigate the different aggregate formations among strains, the frequency of cells with different aggregates was calculated and sorted by the number or size of the dot(s). Generally, [PSI+]s carried by BY4741 strain backgrounds had single foci with evenly distributed small and large size dots (Fig. 2C). Importantly, no remarkable differences were found among the Ura^+^ [PSI+] and Ade^+^ [PSI+] isolates or among Ade^+^ [PSI+] variants with different *ura3–14* nonsense suppression phenotypes. However, [PSI+]s in 74–D694 strain backgrounds showed multiple foci at relatively high frequencies, with a combination of small and large dots (Fig. 2C). Ura^+^ [PSI+] isolates with a strong Ade^+^ phenotype had a relatively high frequency of single dots per cell, whereas Ura^+^ [PSI+] isolates with a weak Ade^+^ phenotype had a relatively high frequency of multiple dots per cell.

### [PSI+] inducibility by [*PIN*+] from different strain backgrounds

**Fig. 3.**
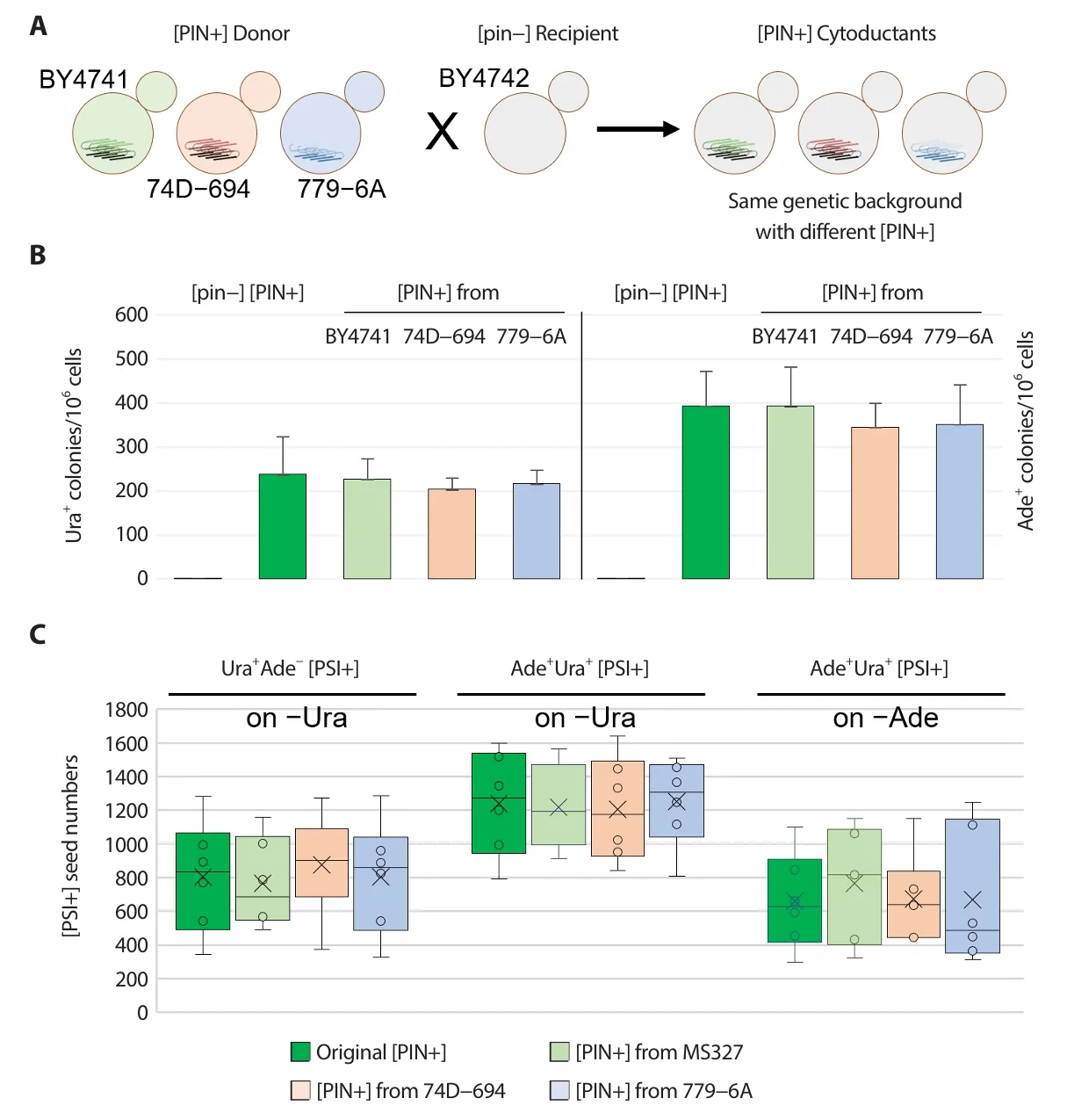
Comparative analysis of [PIN+] prion introduced from BY4741, 74D−694 and 779−6A strain. (A) All the [PIN+]s from different sources was introduced by cytoduction into [pin−] strain (BY4742/MS173). Then the [PIN+] in stains was transferred back into MS327 [pin−] strain (previously made [pin−] by GdnHCl) by cytoduction. [PSI+] was generated by overproduction of Sup35 NM in 2% (wt/vol) raffinose, 2% (wt/vol) galactose minimal medium. Cells were plated on −Ura or −Ade plates, and arising Ura^+^ and Ade^+^ ([PSI+]) clones were tested for GdnHCl curability using transient growth on plates containing 5 mM guanidine. (B) The efficiency of [PSI+] inducibility of [PIN+]s. MS327 [pin−] strain, original [PIN+] carrying strain and [PIN+] from corresponding strain were shown on the bar (left to right). (C) The seed numbers of [PSI+]s isolated in strain carried with different sources of [PIN+]s (n = 12). The seed numbers of Ura^+^ or Ade^+^ [PSI+] isolates with different auxotrophic phenotypes confirmed by other reporter system were confirmed on −Ura media and −Ade media. Arising Ura^+^ or Ade^+^ ([PSI+]) clones were tested for GdnHCl curability using transient growth on plates containing 5 mM guanidine.

The characteristics of certain prion variants can be altered by genetic alterations such as the deletion or overproduction of prion-related genes, even in isogenic genetic backgrounds, or changes in genetic backgrounds. For example, the deletion of Hsp90 and its cochaperones was reported to alter [*PIN*+] prions in isogenic strains, leading to a variety of changes, such as alteration in [PSI+] prion induction efficiency, cellular aggregate formation, and genetic dominance of prion variants (Lancaster et al., 2013). To compare the characteristics of [*PIN*+] prion variants in each strain, [PSI+] inducibility among [*PIN*+] strains from different backgrounds and the phenotypes of the resulting [PSI+] isolates were analyzed. Each [*PIN*+] prion variant was transferred into BY4741 [psi−][pin−] WT recipient strain prepared by transient GdnHCl treatment, and [PSI+]s were then induced in such WT strains with different [*PIN*+] variants from BY4741, 74–D694 and 779–6A strain (Fig. 3A).

The frequency of Ura^+^ [PSI+] clones induced by overproduced Sup35p NM was approximately 200 per 10^6^ cells, and that of Ade^+^ [PSI+] clones was approximately 350 per 10^6^ cells (Fig. 3B, Table 3). The nonsense suppression phenotype of Ura^+^ [PSI+] isolates in WT strains with [*PIN*+] variants from different sources was consistently Ade– on media lacking adenine (Table 1). Ade^+^ [PSI+] clones showed different *ura3–14* suppression phenotypes depending on the isolates (Ura^+^: 4–5/12, mostly Ura^+^: 1–3/12, and Ura^–^: 4–6/12) (Table 1).

The number of Ura^+^ and Ade^+^ [PSI+] clones was determined as described above. Ade^–^ showing Ura^+^ [PSI+] clones had approximately 500–1,000 seeds per cell on –Ura media, and Ura^+^ showing Ade^+^ [PSI+] clones harbored 1,000–1,500 and 400–1,000 seeds per cell when detected on –Ura and –Ade media, respectively (Fig. 3C). These results suggest that all the [*PIN*+] variants from different strains have the same [PSI+] inducibility, resulting in [PSI+] isolates with very similar seed numbers.

## Discussion

In yeast cells without the [PSI+] prion ([psi–] state), Sup35p (eRF3) is soluble, fully functional, and works with Sup45p (eRF1) to efficiently recognize stop codons (UAA, UAG, and UGA) in mRNA, leading to the precise termination of protein synthesis. When Sup35p is converted to the [PSI+] prion form, it misfolds and aggregates into insoluble amyloid fibrils. This sequestration of functional Sup35p leads to a decreased availability of active Sup35p for an efficient translation termination. Therefore, the ribosome often reads through stop codons, allowing translation to continue past where it should normally stop. This is known as nonsense suppression (Paushkin et al., 1997; Stansfield et al., 1995). This nonsense suppression effect by [PSI+] is what makes [PSI+] easily detectable, primarily through nonsense mutation-containing alleles, such as *ade1−14*, *ade2−1*, and *ura3−14*.

**Fig. 4.**
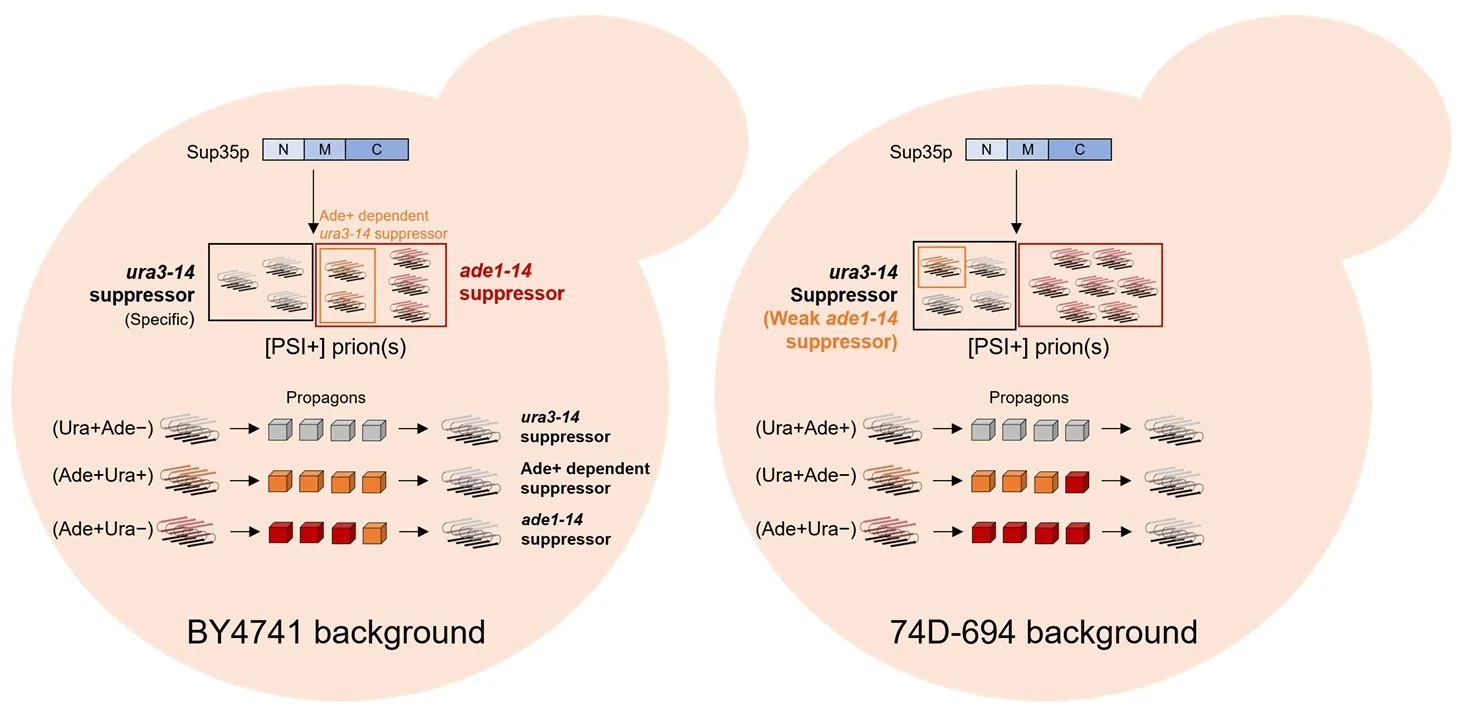
Detection system− and strain− dependent diversity of yeast [PSI+] prion. During the conversion of Sup35p to [PSI+] prion(s), prion amyloid with different characteristics were appeared. In BY4741 strain background (Left), prion amyloids with grey and red color are able to suppress *ura3−14* and *ade1−14* in a specific manner, respectively, while amyloid of orange color can be detected by both systems. Cubes with different colors designate the prion propagon from amyloid of same color and are showing same phenotype with each amyloid. In 74D−694 strain, the detectability appears in reverse: *ura3−14* allele is generic detection system but *ade1−14* allele is specific detection system.

In this study, we investigated how de novo [PSI+] prion generation, detection, and characteristics vary across common laboratory yeast strains (BY4741, 74D−694, and 779−6A) using different nonsense suppression alleles. While [PSI+] could be induced in BY4741 and 74D−694 (with higher frequency in 74D−694 via Ade^+^ selection), the 779−6A strain did not yield true [PSI+] prions that could be detected through Ura^+^ phenotypes. Notably, [PSI+] clones isolated using one detection system (e.g., *ura3−14* for Ura^+^ selection) often displayed different nonsense suppression phenotypes when tested with another allele (e.g., *ade1−14* for Ade^+^) (Fig. 4), revealing a complex relationship between prion variants, strain backgrounds, and reporter systems. Unexpectedly, we found that the frequency of [PSI+] prion variants generated by Sup35p NM overexpression was higher than that previously obtained using a single detection system. Although the sum of [PSI+]s detected by the two systems may not capture every aspect, overexpression of Sup35p NM induced the generation of more diverse and numerous [PSI+] prion variants than anticipated.

Quantitative analysis of prion seed numbers and aggregate structures further highlighted these discrepancies, showing that seed counts varied widely depending on the detected allele and phenotype, and the aggregate morphology differed between BY4741 (single focus) and 74D−694 (multiple foci) (Fig. 4) backgrounds. Interestingly, despite these phenotypic differences, the underlying [*PIN*+] prion variants from different strains neither affected [PSI+] inducibility nor the resulting prion seed numbers of [PSI+] variants when transferred to a common [psi−][pin−] background, suggesting that the observed variability primarily stems from the specific detection system and strain context rather than the initial [*PIN*^+^] seed itself.

### Why do different detection systems lead to different [PSI+] prion variant phenotypes?

**Nature of the reporter system and its requirements:** In all strains, Ura^+^ and Ade^+^ [PSI+] isolates showed significant differences regarding generation frequencies, phenotypes, and propagon number (Fig. 1, Tables 3 and 4). For example, the frequency of appearance of Ura^+^ [PSI+]s was relatively lower than that of Ade^+^ [PSI+]s even under the same induction conditions. Furthermore, Ura^+^ [PSI+]s were not Ade^+^ at all, but Ade^+^ [PSI+]s showed complex phenotypes that were even mixed with Ura^+^ or Ura^−^ phenotypes in certain isolates (Table 4).

The *ade1−14* nonsense allele carries a TGG-to-TGA mutation at codon 244 in the *ADE1* gene, while the *ade2−1* nonsense allele features a GAA-to-TAA mutation at codon 64 in the *ADE2* gene, and they both introduce premature stop codons, compared with each of the WT alleles (Cox, 1965; Inge-Vechtomov et al., 1988; Liebman et al., 1975; Roman, 1956). Unlike other characterized mutations on *ADE* genes, the *ura3−14* allele was engineered, and an 81 nucleotide region (codons 234−254) from the *ade1−14* allele was inserted between the *PGK1* promoter and the *URA3* gene by in−frame insertion mutagenesis (Manogaran et al., 2006). These structural differences in nonsense alleles may lead to different mRNA contexts surrounding the stop codon; alternatively, the proteins they encode (ADE1/ADE2 vs. URA3) may have different levels of tolerance to stop codon readthrough (Chernoff et al., 2002; Fitzpatrick et al., 2011; Namy et al., 2003). For example, a partial readthrough that produces just enough functional Ura3 protein to allow growth on −Ura media might not be enough to produce functional Ade1/Ade2 proteins to grow on −Ade media, meaning that the different thresholds of nonsense suppression due to a certain [PSI+] prion variant might induce "strong enough" readthrough for one reporter but not for another.

Proteins encoded by *ADE1*/*ADE2* and *URA3* play different roles in the cell. Even if both reporter genes are affected by nonsense suppression, the physiological consequences or the amount of functional protein required for a noticeable phenotype can vary. The specific functions of each of these genes may have different effects on [PSI+] prion detectability of nonsense alleles during the selection process.

The *ade* alleles used in this study are typically integrated into the chromosome, while the *ura3−14* allele is on a plasmid. This might lead to differences in gene dosage or chromosomal context, which can influence expression levels and, consequently, the sensitivity of the detection system. Additionally, despite being designed with a yeast centromeric region (*CEN*), multiple studies indicate that the actual copy number of *CEN* plasmids can be “higher than single copy,” and often in the range of three to five copies per cell (Flagg et al., 2019). Thus, the physical location of the chromosome and copy number of pM6 may affect detectability.

**Prion variant/strain conformation:** The core reason for prion variant diversity is that Sup35p can misfold into many distinct amyloid conformations. Each of these conformations acts as a unique template for further Sup35p aggregation (Frederick et al., 2014; Ohhashi et al., 2018; Toyama et al., 2007). These different Sup35p conformations, even if they all lead to some level of nonsense suppression, might do so with varying efficiencies. For example, the "strong" [PSI+] variant (L2892) might cause a highly efficient readthrough phenotype, affecting multiple nonsense reporters, showing stable Ade^+^ and evenly distributed Ura^+^ and Ura^−^ phenotypes. In contrast, the "weak" variant (L2885) might only cause enough readthrough for the most sensitive reporter, showing Ade^+^ and a uniform Ura^−^ phenotype (Table 4). Moreover, stable Ade^+^ [PSI+]s in BY4741 can be categorized by their sensitivity to the *ura3−14* allele-based reporter as Ura^+^ (6/18), Ura^−^ (6/18), and mostly Ura^−^ (6/18) (Table 4). Taken together, these results suggest that amyloid conformational diversity might lead to different translational impacts on other detection reporter systems, resulting in different sensitivities.

**Strain−specific genetic background (host factors):** The stringencies of the three reporter systems were completely different among the strains used in this study. An antithetic operation between BY4741 and 74D−694 was observed, whereas the *ura3−14* allele-based reporter system not working at all in 779−6A (Table 3). In addition, subtle differences in Sup35p aggregation were observed between the two strains tested. In the 74D−694 strain, multiple foci were more frequently detected by galactose-induced Sup35 NM GFP expression (Fig. 2). In addition, different strains can exhibit variations in the expression levels or activities of cellular chaperones (such as Hsp104, Hsp70s, and Hsp40s) and other anti-prion factors (such as Btn2p, Cur1p, NMD factors, Siw14p, and RAC components) (Gorkovskiy et al., 2017; King, 2025; Son and Wickner, 2018; Wu et al., 2023). Chaperones are crucial for prion propagation, the fragmentation of aggregates (which create new "seeds"), and even curing. Differences in these host factors can modulate the apparent strength or stability of prion variants, leading to different phenotypic readouts. This may also affect the mechanism of Sup35p aggregation (e.g., the number of aggregates per cell, size, stability, and interaction with chaperones) in different strains. These subtle structural differences can lead to different levels of functional Sup35p sequestration and, thus, different degrees of nonsense suppression in different strains.

BY4741 is a direct descendant of the S288C lineage, the first strain whose genome was fully sequenced. Strains 74D−694 and 779−6A have been extensively used and are particularly prominent in the field of prion research. Direct comparisons of strain genomes have revealed that even closely related laboratory strains can harbor single nucleotide polymorphisms (SNPs) or other genetic variations that might subtly or significantly influence protein folding, quality control, translation, or the stability of prion aggregates (Drozdova et al., 2016; Fitzpatrick et al., 2011). These differences in "modifier genes" could directly alter the phenotypic expression of prion conformations or affect adenine or uracil biosynthesis pathways independently of the nonsense suppression, indirectly influencing the reporter phenotype in different strains.

### Closing remarks

In essence, the apparent differences in prion variants among different detection systems are not due to changes in the underlying prion proteins themselves, but rather because: 1) the reporter systems have different sensitivities or functional requirements; 2) specific prion conformations (variants) have inherent differences in their ability to induce nonsense suppression, and 3) the genetic background of a host strain can modify the expression or propagation efficiency of prion variants, thereby altering the observed phenotype. This complexity underscores the challenge and importance of precisely characterizing prion variants, and highlights why researchers often use multiple detection methods and different genetic backgrounds to gain a comprehensive understanding of prion biology.

## Figures and Tables

**Table 1.** Strains and plasmids used in this study

Strain	Genotype	Reference
MS327/BY4741	*MATa ura3 leu2 his3 met15 ade1-14kar1* [psi−][PIN+]	Son and Wickner (2018)
MS207/74D−694	*MATa ura3 leu2 his3 trp1 ade1-14 kar1* [psi−][PIN+]	Bradley et al. (2002)
MS1009/779−6Aa	*MATa ura3 leu2 his3 trp1 ade2-1 SUQ5 *(*SUP16*) *kar1* [psi−][PIN+]	Jung et al. (2000)
MS80/L2892	*MATa ura3 leu2 his3 trp1 ade1-14 kar1* [PSI+S][pin−]	Bradley et al. (2002)
MS81/L2885	*MATa ura3 leu2 his3 trp1 ade1-14 kar1* [PSI+W][pin−]	Bradley et al. (2002)
MS173	*MATα ura3 leu2 his3 lys2 ade1-14 kar1* [psi−][pin−]	Son and Wickner (2018)
MS1075	MS327 [PIN+] from MS327	This study
MS1076	MS327 [PIN+] from MS207/74D−694	This study
MS1077	MS327 [PIN+] from MS1009/779−6Aa	This study
**Plasmid**	**Description**	**Reference**
pM6/p1520	*pCEN LEU2 URA3-14 P_GAL1_:SUP35 NM*	Son and Wickner (2018); Wickner et al. (2017)
pM107	*pCEN KanMX P_ADH1_:SUP35 NM GFP*	Nakayashiki et al. (2005)

**Table 2.** The frequency of de novo [PSI+] generation in BY4741, 74D−694, and 779−6A strain

Strain	Total Ura^+^ colonies per 10^6^ cells	Total Ade^+^ colonies per 10^6^ cells
BY4741	191.7 ± 39.6	372.3 ± 69.6
74D−694	135.3 ± 51.3	978.6 ± 84.8
779−6A	^*^17.3 ± 3.5	51.0 ± 11.1

Strains MS327 (BY4741), MS207 (74D−694), and MS1009 (779−6A), used in this experiment, carry their own [PIN+]. For induced [PSI+] generation, strains carrying pM6 with *SUP35 NM* driven by a galactose-inducible promoter were grown 2 days in 2% galactose / 2% raffinose medium at 30°C, and 10^6^ yeast cells were spread on SC plates without uracil or adenine. The presence of nonsense alleles, *ura3-14* on pM6 and *ade1-14* or *ade2-1* on chromosome (integrated), enabled to count colonies which showing nonsense suppressor phenotype. The average number of colonies formed after 5−7 days of incubation at 30°C is shown (the data was collected from three independent experiments were combined). Numbers represent Ura^+^ or Ade^+^ colonies with [PSI+] prion that were confirmed by Guanidine hydrochloride curability.

* In case of 779-6A strain, all the Ura^+^ colonies were unable to confirm the curability indicating that these Ura^+^ colonies were not with [PSI+]. Ura^+^ or Ade^+^ colonies ± SD is shown.

**Table 3.** The nonsense−suppression phenotype of [PSI+] isolates in BY4741, 74D−694, and 779−6A strain

Strain	Ura^+^ [PSI+] isolates on −Ade plate	Ade^+^ [PSI+] isolates on −Ura plate
BY4741	Uniformly Ade^−^ (18/18)	Ura^+^ (6/18)
		Ura^−^ (6/18)
		^a^Mostly Ura^−^ (6/18)
74D−694	^b^Strongly Ade^+^ (3/13)	Ura^+^ (0/18)
	^c^Weakly Ade^+^ (6/13)	Ura^−^ (6/18)
	Ade^−^ (4/13)	^d^Mostly Ura^−^ (12/18)
^e^L2892	N/A	Evenly Ura^+^ and Ura^−^
^f^L2885	N/A	Ura^−^
779−6A	^g^N/A	*Uniformly Ura^−^ (18/18)

Ura^+^ or Ade^+^ [PSI+] isolates in MS327 (BY4741), MS207 (74D−694), and MS1009 (779−6A), listed in Table 2, were streaked for single colonies on prion non−selectable media then replica−plating on the prion−selectable media (−Ura and −Ade) to determine the nonsense−suppression phenotype produced by each of [PSI+] isolates. All the strains carried pM6.

a Mostly Ura^−^ indicates that 80–90% of subclones of corresponding Ade^+^ isolates were Ura^−^.

b Strongly Ade^+^ indicates that Ura^+^ isolates can grow well and showing white color on −Ade media.

c Weakly Ade^+^ means Ura^+^ isolates can grow well and showing pink or slightly red color on −Ade media.

d Mostly Ura^−^ designates 70–90% of subclones of corresponding Ade^+^ isolates were Ura^−^.

e L2892 and

f L2885 are original Ade^+^ isolates showing strong and weak *ade1-14* suppression phenotype (Sue Liebman’s paper).

g N/A stands for not applicable due to none of [PSI+] from Ura^+^ colonies in 779−6A (Table 2).

**Table 4.** The nonsense−suppression phenotype of [PSI+] isolates in BY4741with different [PIN+] from BY4741, 74D−694, and 779−6A strain

Source of [PIN+]	Ura^+^ [PSI+] isolates on −Ade plate	Ade^+^ [PSI+] isolates on −Ura plate
original	Uniformly Ade^−^ (10/10)	Ura^+^ (5/12)
		Ura^−^ (5/12)
		^a^Mostly Ura^−^ (2/12)
BY4741	Uniformly Ade^−^ (12/12)	Ura^+^ (4/12)
		Ura^−^ (5/12)
		^a^Mostly Ura^−^ (3/12)
74D−694	Uniformly Ade^−^ (12/12)	Ura^+^ (4/11)
		Ura^−^ (6/11)
		^d^Mostly Ura^−^ (1/11)
779−6A	Uniformly Ade^−^ (12/12)	Ura^+^ (4/11)
		Ura^−^ 4/11)
		^d^Mostly Ura^−^ (3/11)

Ura^+^ or Ade^+^ [PSI+] isolates in MS327 with different (BY4741), MS207 (74D−694), and MS1009 (779−6A), listed in Table 2, were streaked for single colonies on prion non−selectable media then replica−plating on prion−selectable media (−Ura and −Ade) to measure the nonsense−suppression phenotype produced by each of [PSI+] isolates. All the strains carried pM6.

a Mostly Ura^−^ indicates that 80–90% of subclones of corresponding Ade^+^ isolates were Ura^−^.

b Strongly Ade^+^ indicates that Ura^+^ isolates can grow well and showing white color on −Ade media.

c Weakly Ade^+^ means Ura^+^ isolates can grow well and showing pink or slightly red color on −Ade media.

d Mostly Ura^−^ designates 70–90% of subclones of corresponding Ade^+^ isolates were Ura^−^.

e L2892 and

f L2885 are original Ade^+^ isolates showing strong and weak *ade1-14* suppression phenotype (Sue Liebman’s paper).

g N/A stands for not applicable due to none of [PSI+] from Ura^+^ colonies in 779−6A (Table 2).
